# A comprehensive review in improving delivery of small-molecule chemotherapeutic agents overcoming the blood-brain/brain tumor barriers for glioblastoma treatment

**DOI:** 10.1080/10717544.2019.1616235

**Published:** 2019-05-16

**Authors:** Da Wang, Chao Wang, Liang Wang, Yue Chen

**Affiliations:** a State Key Laboratory of Medicinal Chemical Biology, Nankai University, Tianjin, China;; b Department of Chemistry, Yale University, New Haven, CT, USA

**Keywords:** Small-molecule agents, drug delivery, blood-brain barrier, blood-brain tumor barrier, glioblastoma

## Abstract

Glioblastoma (GBM) is the most common and lethal primary brain tumor which is highly resistant to conventional radiotherapy and chemotherapy, and cannot be effectively controlled by surgical resection. Due to inevitable recurrence of GBM, it remains essentially incurable with a median overall survival of less than 18 months after diagnosis. A great challenge in current therapies lies in the abrogated delivery of most of the chemotherapeutic agents to the tumor location in the presence of blood-brain barrier (BBB) and blood-brain tumor barrier (BBTB). These protective barriers serve as a selectively permeable hurdle reducing the efficacy of anti-tumor drugs in GBM therapy. This work systematically gives a comprehensive review on: (i) the characteristics of the BBB and the BBTB, (ii) the influence of BBB/BBTB on drug delivery and the screening strategy of small-molecule chemotherapeutic agents with promising BBB/BBTB-permeable potential, (iii) the strategies to overcome the BBB/BBTB as well as the techniques which can lead to transient BBB/BBTB opening or disruption allowing for improving BBB/BBTB-penetration of drugs. It is hoped that this review provide practical guidance for the future development of small BBB/BBTB-permeable agents against GBM as well as approaches enhancing drug delivery across the BBB/BBTB to GBM.

## 1. Introduction

### 1.1. Glioblastoma (GBM)

Glioblastoma multiforme (GBM) has been designated by the World Health Organization (WHO) as grade IV glioma or grade IV astrocytoma, and is the most common and lethal primary brain tumor, which is associated with its high resistance to conventional radiotherapy and chemotherapy as well as inevitable recurrence. Due to the infiltrative growth behavior of GBM, complete surgical resection is insufficient to control tumors, resulting in high rates of recurrence which is the primary cause of death. Generally, an annual incidence of 0.6–3.7 per 100,000 individuals for GBM is estimated worldwide (Ostrom, Bauchet, et al., 2014). Despite aggressive surgical resection with postoperative radiotherapy and concomitant chemotherapy, the median overall survival of GBM patients remains 12–15 months following diagnosis (Ostrom, Gittleman, et al., 2014), with less than 5% of people surviving longer than 5 years (Adeberg et al., 2014).

Despite continuous effort directly toward developing new therapeutic approaches as well as novel molecularly tumor-targeted chemotherapeutic agents for better control of malignant glioblastoma, GBM remains essentially incurable attribute to its cellular heterogeneity and drug-resistance nature. Currently, the standard therapeutic regimen for GBM involves maximal feasible surgical resection, followed by postoperative radiotherapy with concomitant and adjuvant temozolomide (TMZ) (Cantanhede & de Oliveira, 2017; Lim et al., 2018). Numerous evidences from clinical trails have demonstrated that, the standard treatment is able to significantly extend the time to recurrence and median survival of the patients who were under 70 years old with newly diagnosed GBM (Davis, 2016). In addition, a number of active chemotherapeutic drugs including the nitrosourea compounds carmustine (BCNU) and lomustine (CCNU) as well as the platinum agents cisplatin and carboplatin, have been considered for GBM treatment as well. Especially, cisplatin and carboplatin were the drugs that have been given as first line agents in patients with GBM in the past. However, the most widely used chemotherapeutic drug is temozolomide (TMZ), an oral small-molecule alkylating agent, which is an imidazotetrazine derivative of dacarbazine. It exhibits cytotoxic effect via preferentially O^6^-methylation of guanine in DNA, leading to G2/M-phase arrest in tumor cells (Pourgholi et al., 2016). TMZ is capable of penetrating into the central nervous system (CNS) and has 96–100% bioavailability (Davis, 2016). In 1999, TMZ was first approved by the U.S. Food and Drug Administration (FDA) for the therapeutic agent against maglinant gliomas. According to preliminary clinical studies, serving as a single agent, TMZ displayed a good response rate of 11% and stable disease in 47% of GBM patients. Further, TMZ was found to be continuously effective with a response rate of 23.8% in patients with GBM in clinical phase II studies (Pourgholi et al., 2016; Cantanhede & de Oliveira, 2017). In comparison to several chemotherapeutic agents, such as BCNU, CCNU, and platinum drugs, TMZ was the best option that improved the median survival time in GBM patients (Hardell et al., 2009). Moreover, TMZ chemotherapy was commonly included in combination treatment with radiotheapy. In a large phase III trail involving 575 patients with GBM, compared with radiotherapy alone, concurrent radiation and TMZ treatment improved the median overall survival from 12.1 to 14.6 months while the 2-year survival rate was increased from 10% to 27% (Khosla, 2016; Lim et al., 2018). The concomitant radiotherapy with TMZ chemotherapy have many advantages including minimal additional toxicity (Lim et al., 2018) and improved radio-sensitivity of tumor cells (Chamberlain et al., 2007). Furthermore, radiotherapy has been reported not only to facilitate spontaneous conversion of TMZ into its active component in the brain, but also to confer TMZ with a good permeability across the blood-brain barrier (BBB) (Lim et al., 2018).

The accumulation of TMZ in tumors is still disappointing, although it is capable of penetrating the BBB. TMZ has been found to be one of the substrates of P-glycoprotein (P-gp), an important efflux pump which locates on the apical membrane side of endothelial cells forming BBB and serves as a maintainer of the integrity and the polarity of BBB (Goldwirt et al., 2014). Due to the presence of P-gp at the BBB, only 20% of TMZ regarding a systemic dose is able to enter the cerebral parenchyma (Ostermann et al., 2004). Additionally, BBB also accounts for the limited efficacy of other chemotherapeutic agents in GBM, such as etoposide, irinotecan, vincristine, and cisplatin. Undeniably, BBB and blood-brain tumor barrier (BBTB) greatly contribute to the therapy resistance in GBM. Therefore, more and more efforts deserve to be devoted to developing novel therapeutic strategies to surpass or to bypass these obstacles, in order to improve the GBM treatment.

### 1.2. BBB

**Figure 1.**
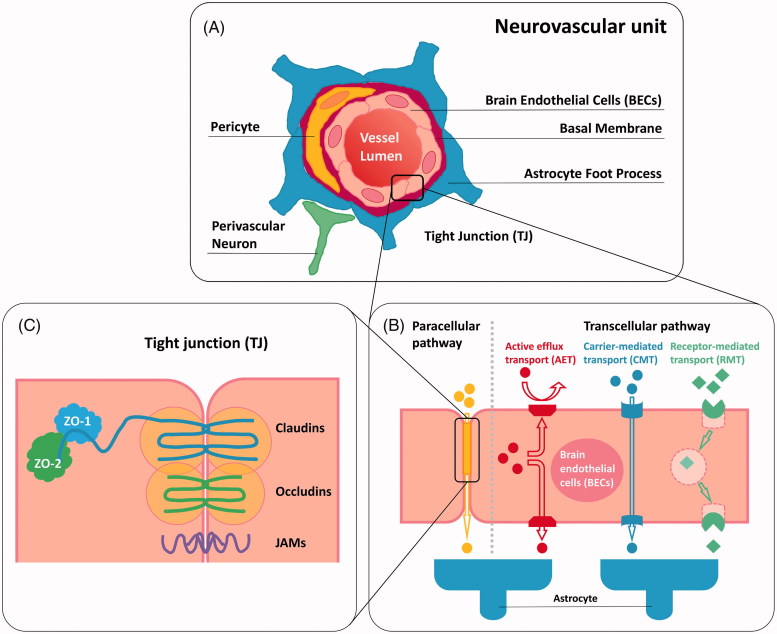
Schematic representation of (A) neurovascular unit; (B) paracellular transport pathway and transcellular transport pathway of BBB; (C) tight junction (TJ) associated components.

Despite ongoing development of novel chemotherapeutic agents against GBM, achieving effective drug delivery into the tumors remains a big challenge, which results in a poor prognosis for this malignant cancer. The key hurdle to effective treatments is the BBB. The BBB serves as an anatomical and physiological barrier which is crucial for protecting the CNS from the potentially harmful substances, such as pathogens and neurotoxic compounds circulating in the blood stream. In addition to its protective function, the BBB modulates the metabolic exchanges between the brain parenchyma and blood for maintaining CNS fluid homeostasis, which is essential for normal function of the brain. In essence, the BBB is composed of non-fenestrated brain endothelial cells (BECs) of the capillary wall, which is surrounded with pericytes, astrocytes, perivascular neurons, a basal membrane, and extracelluar matrix, forming the highly organized neurovascular unit (Figure 1(A)) (Abbott et al., 2006; Deeken & Löscher, 2007; Winkler et al., 2011). Pericytes share a basal lamina with endothelial cells and play a critical role in the maintenance of the structural integrity of the BBB (Obermeier et al., 2013). The basement membrane, which ensheaths both pericytes and endothelial cells, is surrounded with the plasma membranes of astrocytic end-feet, providing biochemical support to the endothelium and contributing to the barrier properties of the BBB (Abbott et al., 2006; Alvarez et al., 2013). Furthermore, astrocytes are contiguous with neurons, which allows for the communication between brain vasculature and neuronal metabolic demand (Winkler et al., 2011).

The human brain contains over 100 billion capillaries with a total length of ∼400 miles, which endows the BBB with a complete surface area of 20 m^2^ (Pardridge, 2005). Generally, small or gaseous molecules such as H_2_O, O_2_, and CO_2_ are able to freely traverse the BBB via passive diffusion, as can some small highly liposoluble/hydrophobic molecules (Wong et al., 2013; Azad et al., 2015). Contrarily, the transport of most of hydrophilic compounds and macromolecules, such as immunoglobulins, are tightly restricted by the BBB (Rodriguez et al., 2015; Weidle et al., 2015; Miranda et al., 2017a). These substances that cannot readily penetrate through the plasma membranes of BECs have to take an active transport pathway across the BBB (Weidle et al., 2015). The endothelial barrier defense system involves different protective mechanisms including transport barrier (paracellular and transcellular), enzymatic barrier, and immunologic barrier (Hendricks et al., 2015), in which the transport barrier plays a predominant role in the entire brain shielding system. In the strict sense, the major barrier function of the BBB is endorsed by the tight junctions (TJs) regulating the paracellular transport, and by the active efflux transporters (AETs) that mainly prevent brain entry of many anti-tumor drugs along transcellular transport pathway (Figure 1(B)). TJs appear as a branching network of sealing strands that effectively stitch adjacent endothelial cells together into a barricade (Figure 1(C)) (Giepmans & van Ijzendoorn, 2009). Being an important type of cell-cell junction, TJs serve as a highly selective permeable dynamic biointerface between the blood and brain to control the passage of solutes by paracellular trafficking in between BECs (Abbott et al., 2006; Giepmans & van Ijzendoorn, 2009; Daneman & Prat, 2015). Generally, the diffusion of the biological compounds such as proteins and carbohydrates is restricted, whereas the penetration is allowed for hydrophobic molecules. The tight junction associated complex that expressed in the connective region between the BECs comprises a variety of transmembrane proteins such as occludins (1, 2, and 3) and claudins (1, 3, 5, and 12) as well as junctional adhesion molecules (JAM-A, -B and -C) (Wolburg & Lippoldt, 2002; Ballabh et al., 2004; Wolburg et al., 2007; Abbott et al., 2010). Occludins and claudins are two key components of TJs, playing a pivotal role in hampering paracellular diffusion. Claudin-1 and occludins are capable of binding tight junction associated proteins (Cingulin and 7H6) via zona occludens protein 1 (ZO-1), leading to regulation of the tight junction properties (Yu et al., 2005; Stamatovic et al., 2008). Moreover, claudin-3, claudin-5, and potentially claudin-12 are associated with the limitation of the small ions delivery (Wolburg & Lippoldt, 2002).

In addition to preventing paracellular transport, most of molecules, especially a large number of anti-tumor drugs, are not allowed for the transcellular diffusion across the BBB, which is mainly attributed to the presence of AETs. A variety of AETs, including P-glycoprotein (P-gp) (Demeule et al., 2002), breast cancer resistance protein (BCRP) (Huang & Sadée, 2006) and members of multidrug resistance-associated protein (MRP) (Kruh & Belinsky, 2003; Potschka et al., 2003; Zhang et al., 2004; Bronger et al., 2005; Soontornmalai et al., 2006), are present at the luminal side (blood side) of the BBB endothelium. These transporters belong to a superfamily of membrane proteins termed ATP-binding cassette (ABC) transporters which consists of seven different subfamilies (ABCA to ABCG) (Moitra & Dean, 2011). They act as ‘gatekeepers’ to control brain entry of xenobiotic compounds including molecularly targeted and non-targeted agents by actively effluxing these substances into luminal spaces for detoxification (Hartz & Bauer, 2011; Miranda et al., 2017a). The predominant AETs refer to P-gp and BCRP, both of them mainly control brain distribution of many anti-tumor drugs and thus reinforce the BBB (Agarwal et al., 2010; Agarwal, Hartz, et al., 2011; Agarwal, Sane, et al., 2011; Agarwal, Sane, Ohlfest, et al., 2011). P-gp is a 170 kD transmembrane protein which is encoded by the *ABCB1* gene (Ambudkar et al., 1992). It has been extensively studied and reported to confer the tumors with significant multidrug resistance. P-gp resides only on the apical membrane of endothelial cells, which modulates drug transport in a unidirectional manner (Fung et al., 2014). It was already known that almost 60% of all marketed anti-tumor agents could be recognized by P-gp and then were pumped out of the cells back to the blood flow, resulting in reduced therapeutic efficacy and poor brain accumulation of drugs (van Tellingen et al., 2015). In addition to P-gp at the BBB, BCRP and other key efflux transporters such as MRP 1–5 that belong to the ABCC transporter family, play a critical role in restricting brain penetration of a large number of anti-tumor agents (Durmus et al., 2012; Lin, de Gooijer, et al., 2013; Gerber et al., 2014). Moreover, the fact that only a few pinocytic vesicles can be generated in BECs for transcellular transport of molecules is responsible for the limited drug penetration across the BBB as well (Hülper et al., 2013).

Next to the transport barrier, enzymatic barrier and immunologic barrier are another two defense mechanisms that contribute to the BBB. Some neurotoxins and drugs can be degradated by several intra- and extracellular enzymes in the BECs, such as esterase, peptidase, phosphatase, monoamine oxidase, and cytochrome P450, which act as a potentially metabolic hurdle to brain entry of drugs (van Tellingen et al., 2015). Furthermore, immunological responses can be triggered by a variety of BBB supporting cells including microglia and perivascular macrophages, providing a immunologic obstacle to drug delivery (van Tellingen et al., 2015). Taken together, the presence of BBB explains the inefficacy of most of chemotherapeutic agents that otherwise are potent to different cancers when tested for GBM therapy (Agarwal, Sane, et al., 2011; Jue & McDonald, 2016; Karim et al., 2016). Therefore, a potential approach to overcome the low access of anti-tumor agents to the tumor cells has become a major issue in the treatment of GBM.

### 1.3. BBTB

In GBM, the organization and function of the BBB can be impacted due to a series of pathological alterations caused by malignant tumor cells, leading to a tumor-specific delivery pattern of chemotherapeutic agents traversing the BBB. The barrier system in GBM is characterized by excessive vascularization with enhanced BBB permeability, which locates between capillary vessels and brain tumor tissues and is thus termed blood-brain tumor barrier (BBTB) (van Tellingen et al., 2015; Miranda et al., 2017a). The aberrant vascularization and dysfunction of the BBTB are mainly ascribed to over-expression of vascular endothelial growth factor (VEGF) and angiogenesis, which are triggered by tumor-induced hypoxic regions (Plate et al., 2012; van Tellingen et al., 2015). In addition to VEGF, some other pro-angiogenic factors released by GBM tumor cells, such as cytokines, are able to lead to BBB disruption (Oberoi et al., 2016).

Despite the observation of a dysfunctional BBTB in GBM, the degree of breakdown is not homogeneous in the entire barrier system, and an intact BBB occurs in the tumor tissues of many GBM patients (Oberoi et al., 2016). In general, the tumor bulk in GBM can be schematically divided into three major moieties: (i) the tumor core where the normal tissue is completely replaced by neoplastic cells and an enhanced permeability of the blood vessels is present, (ii) the angiogenic forehead which is mainly driven by VEGF expression, (iii) the brain adjacent to tumor, where the invading tumor cells infiltrate into normal brain tissue and the vasculature remains intact (Woodworth et al., 2014; van Tellingen et al., 2015; Dréan et al., 2016). In the past, a more plausible theory used by many is, that accumulation of therapeutic agents in the tumor tissues is favored due to the enhanced permeability and retention (EPR) effect originated from a leaky BBTB (Hendricks et al., 2015). Importantly, however, the GBM is well-known for highly invasive nature and thus does cause widespread proliferation of tumor cells which are located away from the tumor core with a compromised BBTB. These invasive tumor cells favor extending to otherwise healthy cerebral parenchyma with a tight BBB as well as with less marked EPR effect. Chemotherapeutic agents which otherwise are unable to cross the intact BBB can readily reach the main central areas of GBM through the disrupted BBTB, instead of the infiltration areas of GBM with a normal BBB (Sarkaria et al., 2018). Since the less altered BBB region is present in the entire BBB/BBTB system in GBM, a disrupted BBTB actually does not much conduce to drug accumulation in all tumor sites. This might help to understand the disappointing efficacy of most of anti-tumor agents in GBM therapy (Hendricks et al., 2015; Karim et al., 2016) as well as the inevitable recurrence of GBM even after a complete surgical resection of all contrast-enhanced regions of tumor (Hou et al., 2006). Being a key part of the healthy BBB, tight junctions (TJs) effectively prevent the transportation of a variety of chemotherapeutics into the CNS via paracellular pathway. However, the alterations in organization and structure of TJs under GBM conditions have been observed, which is associated with BBB dysfunction. Claudin-3, a critical component of TJs, is lost from the BBB in GBM as reported by Wolburg et al., implying its potential influence on the BBB integrity (Wolburg et al., 2003). Furthermore, a loss of claudin-1 in tumor microvessels as well as down-regulation of claudin-5 and occludin in excessive vascularization have been found, which are responsible for the leakiness of TJs and enhanced permeability of cerebral capillaries, and subsequently, for BBB dysfunction (Liebner et al., 2000). In addition, GBM is commonly featured with over-expression of the drug efflux transporters at the endothelial cells of the BBB/BBTB, especially P-glycoprotein (P-gp) (Leweke et al., 1998). Within the tumor necrotic regions, the protective role of P-gp is grossly reduced attributed to a compromised BBTB. Instead, P-gp activity is shown at the tumor borders where an intact BBB is present (Deeken & Löscher, 2007). This is of clinical significance in GBM treatment following surgical resection since tumor-specific over-expression of P-gp restricts brain entry of chemotherapeutic agents (Cheshier et al., 2009). Moreover, the expression of some drug efflux transporters is found in tumor cells as well, which further contributes to tumor multidrug resistance (Lin et al., 2014).

Taken together, BBB/BBTB prevents a variety of chemotherapeutic agents from reaching the GBM areas in the brain, particularly the tumor infiltration zone, forming a great challenge towards effective treatment of GBM. However, the mechanisms underlying the BBB pathological alterations associated with drug delivery into the CNS remain unclear. Continuous effort is necessary to direct toward understanding the BBB and the BBTB, achieving a complete and accurate pre-clinical evaluation of drug efficacy in GBM.

## 2. Delivery of small-molecule chemotherapeutic agents across the BBB/BBTB

In the following parts, we review in detail the delivery of anti-tumor agents across the BBB/BBTB in GBM, particularly small-molecule chemotherapeutic drugs via paracellular and/or transcellular transport pathways. Brain entry of protein therapeutics such as antibodies, as well as drug delivery mediated by carriers including liposomes, micelles, and nanoparticles are not the focus of this review.

### 2.1. Active efflux transport (AET)-dominated transcellular transport

As outlined above, one of the keys to effective therapy of GBM is achieving adequate accumulation of chemotherapeutic agents in the brain, which refers to their sufficient BBB/BBTB penetration. In general, brain entry of most of the chemotherapeutic agents is blocked due to the presence of endothelial TJs as well as the poor transcytosis (Bauer et al., 2014). A variety of active and specific transporter proteins are commonly expressed in the endothelial cells and involved in the transcellular transport systems, which mediate brain uptake and extrusion of various xenobiotic compounds including chemotherapeutic agents. To date, three main mechanisms of transcellular transport systems have been identified in the BBB (Figure 1(B)), which comprises receptor-mediated transport (RMT), carrier-mediated transport (CMT), and active efflux transport (AET) (Thomsen et al., 2012; Mikitsh & Chacko, 2014). RMT is one of the specific transcytosis mechanisms, which is based on endocytosis from the luminal side and exocytosis from the abluminal side of the endothelium (Lin, de Gooijer, et al., 2013). Some macromolecules including large anti-tumor protein therapeutics can be potentially transported across the BBB via RMT (Pardridge, 2003). Nevertheless, this aspect will not be in detail discussed here. CMT refers to the delivery of specific substances such as sugars, amino acids, organic cations and anions, nutrients and metabolites into the brain, which is mediated by a series of the solute carrier (SLC) transporters expressed at the BBB (Hediger et al., 2004). Glucose transporters (GLUTs), monocarboxylate transporters (MCTs), organic ion (cation and anion) transporters (OCTs and OATs), and nucleoside transporters are the major SLCs involved in CMT (Hediger et al., 2004; Molina-Arcas et al., 2009; Wong et al., 2013; Karim et al., 2016). Reportedly, OAT1, a classical organic anion transporter at the BBB endothelium, was able to modulate the transport of some anti-neoplastic agents, such as methotrexate, doxorubicin, and aclarubicin (Sekine et al., 2000).

The most crucial transcellular transport pathway is active efflux transport (AET) mechanism, which is predominant in the BBB for detoxification. AET is an ATP-driven mechanism that not only hinders brain entry of a large number of xenobiotics including potentially toxic substances and therapeutic agents but also transports the compounds that have crossed the BBB back into the circulation. All active efflux transporters (AETs) have been characterized by nucleotide binding sites that work as a catalytic domain for ATP hydrolysis, which is also reportedly associated with substrate recognition (Hollenstein et al., 2007). The AETs have a wide endogenous and exogenous substrate spectrum. The aforementioned P-gp (ABCB1) is able to identify non-polar and less amphiphilic molecules. Lipids, steroid hormones, and cytokines are the endogenous substrates of P-gp. Moreover, small-molecule chemotherapeutic agents like temozolomide, methotrexate, paclitaxel, anthracyclines, and vinca alkaloids have been proven to be the substrates of P-gp (Demeule et al., 2002; Agarwal, Sane, et al., 2011; Munoz et al., 2014). P-gp is able to actively pump these clinically used drugs out of the tumor region and the BBB/BBTB endothelium back to the brain capillary lumen, which explains their poor response in GBM therapy. Substrates of BCRP (ABCG2) include glutathione and steroid hormones (Mao & Unadkat, 2005) as well as some anti-tumor chemotherapeutic agents like methotrexate, mitoxantrone, and topotecan (Huang & Sadée, 2006), which overlap with those of P-gp (Staud & Pavek, 2005). Next to P-gp and BCRP, MRP refers to the ABCC transporter subfamily, which has 5 members (ABCC1-5) that locates at the BBB (Kruh & Belinsky, 2003; Potschka et al., 2003; Zhang et al., 2004; Bronger et al., 2005; Soontornmalai et al., 2006). MRPs are involved in BBB penetration of more polar molecules, including endogenous metabolites and drugs (Slot et al., 2011). However, both MRP1 (ABCC1) and MRP4 (ABCC4) have overlapping substrate profiles with those of P-gp and BCRP, as suggested by their similar substrate recognition of camptothecins (Lin, Marchetti, et al., 2013) and methotrexate (Kruh & Belinsky, 2003; Sane et al., 2014). MRP1 is also able to recognize vinca alkaloids and anthracyclines which are listed in the substrate spectrum of P-gp as well (Kruh & Belinsky, 2003). Together, most of the AETs effectively clear the small-molecule chemotherapeutic agents that are their substrates by modulating efflux of these drugs from the BBB/BBTB endothelium into the vascular lumen away from the cerebral parenchyma, which have major clinical significance for resistance to chemotherapy in the treatment of GBM. A list of the drug substrates of AETs in relation to GBM is summarized in Table 1.

**Table 1.** Summary of approved small-molecule chemotherapeutic agents for the treatment of brain tumors and their substrate status of AETs.

Chemotherapeutic agents	Targeted brain tumor type	Substrate status of AETs (Ref.)
Temozolomide (TMZ)	GBM; primary central nervous system lymphoma^a^; refractory anaplastic astrocytoma^a^	ABCB1 (Goldwirt et al., 2014)
Lomustine (CCNU)	Grade III glioma; medulloblastoma	No (Dréan et al., 2016)
Carmustine (BCNU)	GBM	No (Hardell et al., 2009)
Procarbazine	Grade III glioma; oligodendrogliomas^a^; primary central nervous system lymphoma^a^	No (Azad et al., 2015)
Cisplatin	Medulloblastoma	ABCC2, ABCC6 (Gerber et al., 2014)
Carboplatin	GBM	No (Dréan et al., 2016)
Topotecan	GBM	ABCB1 (de Vries et al., 2007), ABCG2 (Huang & Sadée, 2006)
Methotrexate	Central nervous system lymphoma^a^	ABCB1 (Agarwal, Sane, et al., 2011), ABCG2 (Staud & Pavek, 2005), ABCC4 (Sane et al., 2014)
Doxorubicin	Neuroblastomas^a^	ABCB1, ABCG2 (Oberoi et al., 2016)
Etoposide	GBM; neuroendocrine tumors^a^	ABCB1 (Dréan et al., 2016)
Irinotecan	GBM	ABCB1 (Goldwirt et al., 2014), ABCG2 (Oberoi et al., 2016)
Vincristine	Grade III glioma; medulloblastoma	ABCB1 (Azad et al., 2015)

^a^Associated conditions with respect to targeted brain tumor types collected from the public data sources of drugbank (https://www.drugbank.ca/drugs).

### 2.2. BBB/BBTB permeation of approved small-molecule agents for GBM

**Figure 2.**
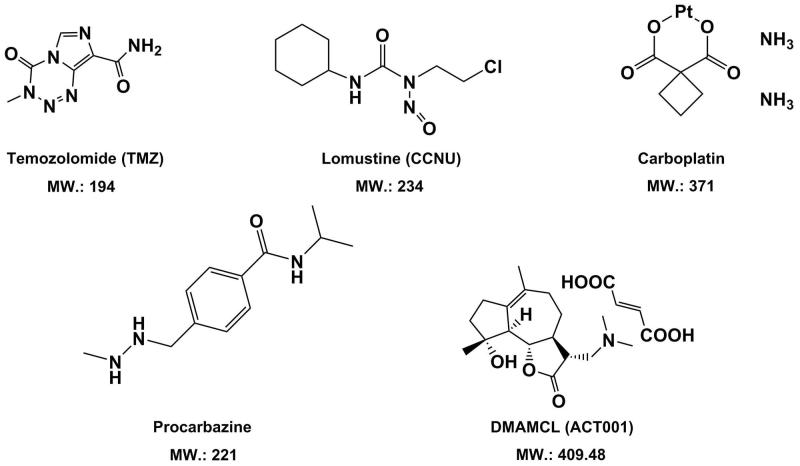
Chemical structures and molecular weight of small-molecule chemotherapeutic agents with good BBB/BBTB permeability in treatment of GBM.

Till now, most curative chemotherapeutic agents with better BBB/BBTB passage for GBM therapies are temozolomide (TMZ) (Reyderman et al., 2004; Portnow et al., 2009; Goldwirt et al., 2014), lomustine (CCNU) (Taal et al., 2014), procarbazine (Sekine et al., 2000; Weidle et al., 2015), and carboplatin (Jacobs et al., 2010; Weidle et al., 2015) (Figure 2). TMZ is to date the most frequently used and the FDA-approved chemotherapeutic agent for GBM treatment (Fernandes et al., 2019). In human brain adjacent to tumor, TMZ displayed a brain/plasma ratio of 18% and a cerebrospinal fluid (CSF)/plasma ratio of 20–40% (Portnow et al., 2009; Goldwirt et al., 2013). On an *in vivo* model of mice, maximal exposure of TMZ in plasma and brain was almost reached concomitantly, namely, the close *T*_max_ for both cases, indicating good BBB/BBTB penetration (Reyderman et al., 2004). However, the expression level of ABCB1 at the BBB/BBTB can affect brain uptake of TMZ, strongly suggesting that TMZ is one of the ABCB1 substrates (Reyderman et al., 2004). Compared with ABCB1, the role of ABCG2 on transport of TMZ across the BBB/BBTB is negligible. In addition, higher tumor accumulation of TMZ is partially attributed to the enhanced permeability and retention (EPR) effect resulted from a less intact BBB (Zhou & Gallo, 2009). CCNU is another chemotherapeutic drug which has been currently tested in clinical trial dedicated to GBM patients (Sepulveda-Sanchez et al., 2015). CCNU exhibited a lower brain/plasma ratio of 20% in rats compared with 22–41% for TMZ, although it is not involved in the substrate spectrum of any ABC transporter (Ostermann et al., 2004; Azad et al., 2015; Dréan et al., 2016). By contrast, procarbazine and carboplatin revealed lower or medium BBB/BBTB passage (Jacobs et al., 2010; Dréan et al., 2016) and higher CNS toxicity (Newton, 2012). The clinical efficacy of them for treatment of brain tumors was still under investigation (Owonikoko et al., 2014).

What most worth mentioning is, that a novel small-molecule chemotherapeutic agent against glioblastoma, DMAMCL (also registered as ACT001), which is featured with a framework of sesquiterpene lactone, has been newly discovered (Zhang et al., 2012; An et al., 2015). Reportedly, it revealed up to 88% tumor inhibition effect in glioblastoma murine models (An et al., 2015). Notably, ACT001 displayed high permeability across the BBB/BBTB *in vivo*. As a novel anti-GBM drug, ACT001 has currently entered in Phase I clinical trials in Australia (trial ID: ACTRN12616000228482) and received the orphan drug designation for GBM from both the FDA in the USA (https://www.accessdata.fda.gov/scripts/opdlisting/oopd/detailedIndex.cfm?cfgridkey=610217.) and the EMA in Europe.

An empirical approach to simply estimate BBB permeability of small-molecule anti-tumor agents, namely ‘the rule of 5’, has been developed by Lipinski in 2001 (Lipinski et al., 2001). To date, this criteria has been still widely applied for predictions of drug-ability and increasingly served as a useful tool for BBB-permeable screening of compounds in drug discovery, although it is not always in line with the experimental data. Herein, instead of going to in detail elucidate the rule of 5, we would introduce four parameters that are considered to be globally associated with BBB penetration, which are molecular weight (MW), Log P, the number of hydrogen bond donors and the number of hydrogen bond acceptors (Lipinski et al., 2012). Better BBB crossing is possible for a new compound when its MW is less than 500 Da; the Log P is below 5; the sum of OH and NH groups (hydrogen bond donors) and the sum of oxygen and nitrogen (hydrogen bond acceptors) are less than 5 and 10, respectively. Due to a cutoff value of 5 or a multiple of 5 for each of the four parameters, it is called ‘the rule of 5’ and is convenient in application. Lipinski’s criteria can also be further simplified, where the limitation of the number of hydrogen bond acceptors is omitted. The rule of 5 has successfully predicted BBB/BBTB passage *in vivo* of the aforementioned chemotherapeutic agents against GBM, as shown in Table S1 in Supplemental Material. For instance, TMZ possesses the lowest experimental Log P value of −2.8 compared with other drugs, indicating the best penetration across the BBB/BBTB. In addition to the rule of 5, rotatable bond count and polar surface area (PSA) of a molecule are another two parameters related to BBB permeability of drugs as well. In general, molecules with more than 10 rotatable bonds are usually believed to have poor BBB permeability. All the drugs shown in Table S1 have rotatable bond count either below or equal to 5. The polar surface area (PSA) refers to the overall surface over all the polar atoms of a molecule, including oxygen, nitrogen, sulfur, phosphorus as well as the attached hydrogens. For a new drug candidate against brain tumors, permeating the BBB/BBTB into the CNS is favored when the PSA is less than 0.90 nm^2^ (And & Pennington, 2006). However, it is still controversial since the prediction from the PSA is not all the time in consistence with that of Lipinski’s criteria. As seen in Table S1, the PSA of TMZ is 1.06 nm^2^ which is greater than 0.53 nm^2^ of procarbazine and 0.62 nm^2^ of CCNU, implying that TMZ is less able to traverse the BBB/BBTB. Instead, the rule of 5 predicts that TMZ is more readily to cross the BBB/BBTB than procarbazine and CCNU, which is also in agreement with the reported experimental data *in vivo* (Jacobs et al., 2010; Azad et al., 2015; Dréan et al., 2016). As for the new small anti-GBM agent ACT001, a physio-chemical profile that hits the limitations of both Lipinski’s rule and PSA is shown (see Table S1 in Supplemental Material) and a convictive BBB/BBTB permeation has been observed in murine models (An et al., 2015). Apart from the molecules displayed in Figure 2, the capacity of penetrating the BBB/BBTB for many other small anti-tumor agents can be estimated by Lipinski’s criteria coupled with the PSA parameters as well. Methotrexate (454 Da) and doxorubicin (544 Da) are two well-known agents which bear a small molecular size of ∼500 Da and have been used as mono-therapy in GBM patients (Newton, 2012). Based on Lipinski’s criteria, methotrexate and doxorubicin are predicted to be poor at permeating the BBB/BBTB, as suggested by 12 hydrogen bond acceptors for both (see Table S1 in Supplemental Material). Their large PSA of greater than 2 nm^2^ indicating poor lipophilicity also support the estimation of the rule of 5. And these predictions are consistent with the experimental data *in vivo* (Van et al., 1999; Westerhout et al., 2014).

### 2.3. BBB/BBTB permeation of drug candidates with anti-GBM potential

**Figure 3.**
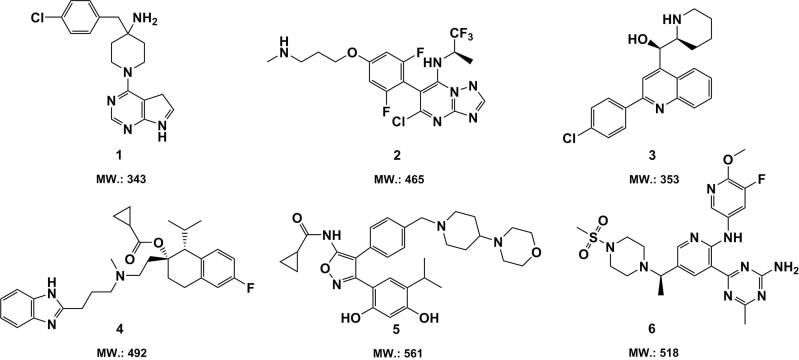
Chemical structures and molecular weight of small candidate molecules (**1**–**6**) with promising anti-GBM activity *in vivo*.

Based on the medicinal chemistry strategies in the development of small molecules for GBM chemotherapy, some anti-GBM drug candidate compounds derived from pyrimidines, quinolines, indazoles, and triazines have been identified in the last decade (Fernandes et al., 2019). These compounds have been demonstrated to effectively inhibit the GBM tumor growth in a panel of models *in vivo*, indicating their better BBB/BBTB permeable potential. Several candidate compounds with superior anti-GBM activity *in vivo* are displayed in Figure 3. (The parameters related with their BBB/BBTB penetration efficacy are summarized in Table S2 in Supplemental Material).

The pyrimidine-based compounds **1**, namely the serine-threonine kinase AKT inhibitor CCT128930, induced remarkable tumor growth inhibition in a human GBM xenograft mice model (Yap et al., 2011). Another pyrimidine-based candidate compound **2** exhibited potent efficacy against human GBM U87MG cells xenografted in mice at a single dose of 25 kg/mg (Beyer et al., 2008). Compound **3**, which contains a quinoline moiety, has been reported to be able to put a brake on the human U3013 GBM cells proliferation in an orthotopic zebrafish model xenografted and meanwhile showed promising bioavailability in mouse tissue (Hammarström et al., 2016). The experimental data implies, that compound **1–3** might have potential to effectively traverse the BBB/BBTB, which is also suggested by the prediction of the rule of 5 (see Table S2 in Supplemental Material). Compound **4–6**, which are respectively derived from indazoles (Kim et al., 2015), triazines (Norman et al., 2012), and oxygen-containing heterocyclics (Chen et al., 2014), displayed significant inhibitive effect on tumor growth of U87MG GBM cells xenografted in mice *in vivo*. Thus, it is possible that they are capable of crossing the BBB/BBTB. However, the parameters shown in Table S2 gave the opposite predictions. Note that compound **6** bears not only a bigger molecular size than 500 Da (518 Da) but also a PSA of 1.50 nm^2^ greater than 0.90^ ^nm^2^ and 12 hydrogen bond acceptors beyond the threshold of 10, which strongly suggests that compound **6** is less lipophilic and thus might be not a potential BBB/BBTB permeable agent. The cases of compound **4–6** state the limits of predictive Lipinski’s criteria.

### 2.4. Drug re-purposing and some BBB-permeable small molecules

**Figure 4.**
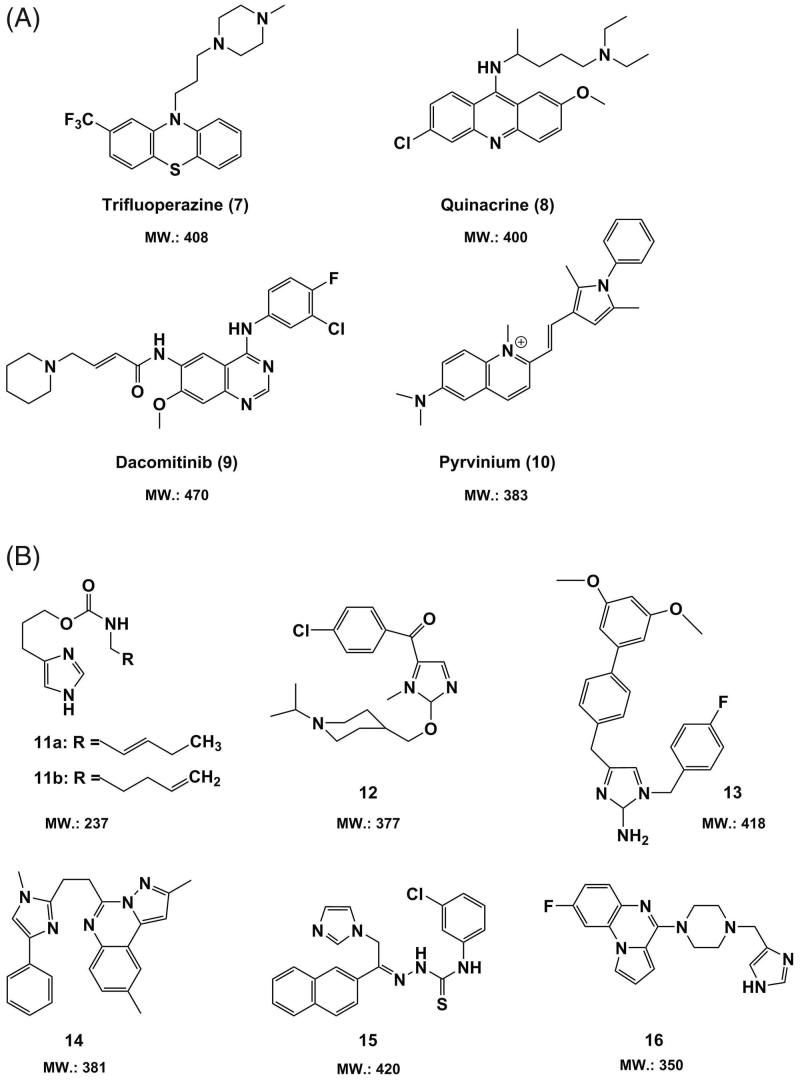
Chemical structures and molecular weight of (A) approved small-molecule drugs (**7**–**10**) re-positioned as anti-GBM agents and (B) imidazolium-based candidate molecules (**11**–**16**) with promising BBB permeability.

Besides to the traditional medicinal chemistry strategies, drug re-purposing approach could accelerate the identification of new small drug candidates with favorable BBB/BBTB permeable properties since many existing drugs have previously revealed acceptable safety and pharmacokinetic profiles (Sminia & Westerman, 2016). Hence, we need to temporarily look away from GBM and re-focus on the already marketed drugs, and the candidate compounds which have been selected for other diseases and are potentially effective in GBM therapy. A library of clinically approved drugs that are routinely employed against several disorders, such as anti-psychotic drugs, could be re-positioned as anti-tumor agents with certain BBB/BBTB permeable potential, as shown in Figure 4(A).

Trifluoperazine (**7**), a typical phenothiazine-based dopaminergic ligand bearing a piperazine cyclic moiety, has been largely used as a dopamine receptor D2 antagonist in schizophrenia treatment. This indicates its better BBB permeability, which is in line with the prediction by Lipinski’s criteria (see Table S2 in Supplemental Material). Trifluoperazine (**7**) displayed a potent anti-GBM activity of 1.34 and 1.85 μM EC_50_ against U3046MG and U3005MG GBM cells, respectively, and was thus identified as a potential anti-GBM drug of traversing the BBB/BBTB (Pinheiro et al., 2017). Quinacrine (**8**), an anti-malarial drug that is derived from acridine, has been demonstrated to be able to induce apoptosis and endoplasmic reticulum stress *in vivo*, which led to cell death of U87 GBM cells xenografted in mice (Golden et al., 2015). The EGFR tyrosine kinase inhibitor dacomitinib (**9**), which has been primarily used against non-small-cell lung carcinoma, significantly inhibited intracranial tumor growth by using mice xenografted with GBM cells, which was evidenced by a reduction of tumor cells proliferation and an induction of apoptosis of tumor cells (Zahonero et al., 2015). The anthelmintic drug pyrvinium (**10**) were reportedly able to abrogate the formation and the recurrence of GBM in GBM xenograft mice (Venugopal et al., 2015). For drug **8–10**, nearly all the parameters related with BBB/BBTB crossing reached the requirements of the rule of 5 (see Table S2 in Supplemental Material), which implies their better BBB/BBTB penetration *in vivo*.

In addition to the re-purposed small-molecule drugs with potential anti-GBM activity, a large number of imidazolium-based drug candidates including anti-histaminic, anti-neuropathic, and serotonin (5-HT)-targeted compounds, were selected for their capability to pass the BBB (Sminia & Westerman, 2016). As shown in Figure 4(B), compound **11a** and **11 b** are two imidazolium carbamates bearing an unsaturated alkyl chain attached to the amino group. Both of them showed highly H_3_ receptor (H_3_R) antagonistic activity *in vivo* and were able to penetrate across the BBB after per-oral administration to Swiss mice (Łazewska et al., 2009). Compound **12** containing a piperidine group was also a highly H_3_R-selective antagonist with a low K_i_ of 3 nM, and displayed remarkable brain entry effect *in vivo* (Ishikawa et al., 2010). Being a potent anti-AD drug candidate, compound **13** which is featured with an active moiety of 2-aminoimidazole, revealed a desired BACE-1-targeted activity (IC_50_=7.4 μM) as well as promising BBB-permeable properties (Chiriano et al., 2012). The imidazole derivative compound **14** served as a highly selective phosphodiesterase 10 A (PDE10A) inhibitor (IC_50_ = 16 nM) for the treatment of Parkinson’s disease (PD). In comparison to its benzimidazole analog, compound **14** revealed an improved BBB penetration efficacy (Wager et al., 2010; Asproni et al., 2011). Compound **15**, a Schiff-base derivative, worked as an effective anti-convulsant candidate agent together with an appropriate liposolubility favoring BBB passage (Caliş et al., 2011). The imidazolium-based compound **16** with a quinoxaline segment was not only a highly 5-HT_3_ receptor-targeted drug candidate but also identified to be a good BBB-permeable agent (Butini et al., 2009). Note that, all the imidazolium derivatives included in Figure 4(B) are predicted to able to pass the BBB but compound **13**, as suggested by the parameters summarized in Table S2 in Supplemental Material.

### 2.5. A simple screening strategy of small BBB/BBTB-permeable drugs

In order to further identify new small-molecule anti-GBM candidate compounds with desired pharmacokinetic profile of BBB/BBTB permeation, the structure-activity relationship (SAR) of the aforementioned drugs and drug candidates i.e. the relationship between their structural features and their BBB/BBTB permeability is described here. First of all, the SAR pointed out that the lipophilic moieties like aromatic rings and alicyclic rings were indispensable for effective BBB/BBTB passage of small molecules. And the substitution of the hydrogen on the benzene ring by chlorine (compound **1**, **2**, **3**, **8**, **9**, **12**, **15**), fluorine (compound **2**, **4**, **6**, **9**, **13**, **16**), methoxyl group (compound **6**, **8**, **9**, **13**), and trifluoromethyl group (trifluoperazine (**7**) was beneficial for improving BBB/BBTB permeability since they were able to boost up the lipid-solubility of drug molecules. Secondly, an active nitrogen posed as a trialkylamine form was present in most of the drugs and drug candidates above mentioned. Such active nitrogen seems most likely to play a vital role in increasing the oil-water partition coefficient of drug molecules and to contribute to their anti-GBM activity. Furthermore, the introduction of a panel of heterocycles containing tertiary amines, including piperidine (compound **1**, **3**, **5**, **9**, **12**), piperazine (compound **6**, **7**, **16**) and morpholine (compound **5**), had a positive influence on BBB/BBTB passage, which was reflected by the SAR as well. Finally, it was worthy to note that, most of the aforementioned drugs and drug candidates reached the limitations given by Lipinski’s criteria with respect to the prediction of their BBB/BBTB penetration. According to all the above-discussed points of the SAR, a simple and optimized screening strategy which combines the advantages of medicinal chemistry approaches and drug re-purposing programs to identify the small-molecule drug candidates with superior BBB/BBTB permeable efficacy could be concluded as follows:

- the molecule possesses appropriate lipid-solubility, which can be estimated by the count of aromatic rings and/or alicyclic rings;
- the molecule is structurally featured with the trialkylamine-based moiety, including dimethylamine and a series of nitrogen-containing heterocycles such as piperidine, piperazine, and morpholine;
- the molecule is predicted to be able to traverse the BBB/BBTB based on the rule of 5.

Following this integrated approach, people could efficiently select small molecules with optimal characteristics of high BBB/BBTB passage from a large number of drug candidates bearing anti-GBM potential. In addition, many public data sources with respect to a variety of bio-pharmaceutic parameters of drugs, such as bio-availability, molecular fingerprint, and neurotoxicity, are also helpful for the identification of BBB/BBTB-permeable drugs (Sminia & Westerman, 2016). Nonetheless, the drug screening process is time-consuming and complex, and it is hard to discover a drug molecule that is inherently able to traverse the BBB/BBTB, given a limited number of small molecules overcoming or bypassing the impermeable obstacle of BBB/BBTB. Therefore, there is a great need to develop a series of approaches and techniques that could enable the opening or the disruption of the BBB/BBTB system to improve drug delivery within the GBM tumor site.

## 3. Strategies for overcoming the BBB/BBTB

Despite many ongoing efforts during the past decades to develop novel chemotherapeutic agents to treat GBM, none of them showed the satisfactory therapeutic effect or led to the enhanced quality of life for GBM patients. The failed attempts are mainly ascribed to the inadequate drug delivery into the tumor tissue because the BBB/BBTB greatly prohibits brain entry of a number of anti-tumor drugs including small-molecule chemotherapeutic agents. To counteract the protective effect of BBB/BBTB, a variety of approaches and techniques have been emerged and become more refined. Basically, these methods were established with the goal of circumventing and modulating the entire BBB/BBTB system, which consists of active efflux transporter (AET) targeted and tight junction (TJ) targeted strategies. The former refers to overcoming active efflux at the BBB/BBTB via structural modification of drug candidates as well as pharmacological inhibition of active efflux transporters (AETs). The TJ-targeted ones involve a series of approaches and techniques applied for opening or disruption of BBB/BBTB, which include chemical-mediated BBB/BBTB disruption, hyper-osmotic BBB/BBTB disruption, and thermotherapy-induced BBB/BBTB opening such as focused ultrasound (FUS), radio-frequency microwaves, and laser interstitial thermotherapy (LITT). Several of them have shown promise and many of the patients with GBM could benefit from these improved drug delivery regimens. In the following section, we will discuss in detail about the current efforts and advances in each of these strategies, excluding nanosystem-based delivery, convection-enhanced delivery, and other delivery systems related to therapeutic interventions.

### 3.1. AET-targeted BBB/BBTB disruption

To reach a drug molecule with enhanced BBB/BBTB permeability, current chemical modification of existing drugs have focused on minimizing their molecular size and increasing their lipophilicity. Given the main defense mechanisms of BBB/BBTB to drug uptake, an anti-GBM drug candidate could be modified to an analog of the ligand against the specific receptor at the BBB/BBTB or could be linked to the ligand to receptor-mediated transport (RMT). For instance, addition of transferrin to drugs can promote their BBB/BBTB passage via transcytosis mediated by transferrin receptor (TfR) (Zhang et al., 2015). The modification of a marketed anti-cancer drug Doxil® (PEGylated liposomes of doxorubicin) with glutathione groups showed a 4.8-fold increased brain/plasma ratio in comparison to Doxil^®^ alone in preclinical studies (Birngruber et al., 2014; Gaillard et al., 2014). Lipid carriers like fatty acids can also be employed to improve drug penetration through the BBB/BBTB. Small drug paclitaxel acquires increased BBB/BBTB permeability by covalent conjugation of N-docosahexaenoic acid (DHA) (Bradley et al., 2001). In addition to the biochemical modifications using the ligand to RMT, structural refinement of drugs to diminish their affinity to AETs at the BBB/BBTB greatly contributes to their brain entry, given that a number of anti-tumor drugs especially molecularly targeted agents have proven substrate liability for both P-gp (ABCB1) and BCRP (ABCG2) (Oberoi et al., 2016). BMK120, a phosphatidylinositol-3 kinase (PI3K) targeted inhibitor, displayed not only superior BBB/BBTB penetration as a consequence of structural modification of minimizing its affinity to AETs but also promising effect in GBM models *in vivo* (Wen et al., 2012). The best approach to overcome active efflux of anti-tumor drugs is using pharmacological inhibitors of AETs. Since most agents are substrates of both P-gp and BCRP (Table 1), inhibition of both AETs should be mandatory to achieve adequate brain uptake of drugs (van Tellingen et al., 2015). A number of inhibitors including cyclosporin A, valspodar, elacridar, and tariquidar are able to modulate the activity of both P-gp and BCRP in multiple pre-clinical models (Bankstahl et al., 2013). As a representative of the first generation inhibitors, cyclosporin A can effectively reverse the efflux effect of P-gp at the BBB/BBTB and an increased brain distribution of docetaxel was observed in mice (Kemper, Boogerd, et al., 2004). However, direct inhibition of AETs at the BBB/BBTB by using most of the first generation inhibitors did not appear sufficient efficacy *in vivo*. This was attributable to their poor binding affinities, which requires the usage of high doses and thus results in intolerable toxicities (Doyle & Ross, 2003; Bhowmik et al., 2015). The treatment of valspodar, a second generation P-gp inhibitor, was reported to induce 9.08-fold increase in brain uptake of vinblastine in rats (Drion et al., 1996). Similarly, brain accumulation of docetaxel was also enhanced when it was co-injected with valspodar in mice (Kemper, Boogerd, et al., 2004). However, both the first and second generation inhibitors have been met with moderate efficacy and limited safety, which restricts their application in clinic. Accordingly, the third generation inhibitors with high affinity to AETs and acceptable tolerability have been developed. Currently, elacridar and tariquidar are the two most promising options in all the third generation inhibitors of AETs. Increased brain concentrations of molecularly targeted agents gefitinib (Agarwal et al., 2010) and vandetanib (Minocha et al., 2012) have been observed in mice with co-injection of elacridar. Moreover, as reported by Kemper et al., paclitaxel uptake was improved 5-fold in the brain with co-administration of elacridar (Kemper et al., 2003). Agarwal et al. showed higher brain accumulation of erlotinib by combinatorial treatment of elacridar in an orthotopic rat xenograft model of U87 GBM (Agarwal et al., 2013). Furthermore, elacridar is also able to inhibit P-gp mediated efflux of docetaxel (Kemper, Boogerd, et al., 2004) and topotecan (de Vries et al., 2007) from the brain. Unfortunately, the excess toxicity induced by the concomitant use of elacridar and cytotoxic chemotherapeutic agents like doxorubicin was also present (Planting et al., 2005). Hence, developing an inhibitor with high specificity to AETs as well as suitable tolerability is necessitated for overcoming active efflux of drugs and worth pursuing in the future.

### 3.2. TJ-targeted BBB/BBTB disruption

Apart from the AET-targeted BBB/BBTB breakdown as discussed above, another attempt to facilitate brain entry of small-molecule drugs is opening or disruption of BBB/BBTB using a panel of chemical and pharmacological agents as well as techniques that are TJ targeted. Since the drug paracellular transport is mainly modulated by tight junctions, it is possible that TJ-targeted means favor the passive diffusion of anti-tumor agents across a compromised BBB/BBTB.

Chemical-mediated BBB/BBTB disruption involves exposing the barrier system to a specific chemical agent that is able to alter the BBB/BBTB integrity. A panel of vasoactive compounds like histamine (Oberoi et al., 2016; Miranda et al., 2017b), bradykinin (Azad et al., 2015; Oberoi et al., 2016; Miranda et al., 2017b), alkylglycerols (Erdlenbruch et al., 2003; Hülper et al., 2013), and tumor necrosis factor ɑ (TNF-ɑ)/interferon γ (INF-γ) (Lopez-Ramirez et al., 2012) can make tight junctions (TJs) disrupted through stimulating B2 receptors at the endothelium and thus induce a transiently increased cytosolic Ca^2+^, which leads to opening of BBB/BBTB (Kemper, Verheij, et al., 2004). Bradykinin and its agonist RMP-7 are two agents that have been most widely studied for chemical-mediated disruption approach. Bradykinin can selectively target B2 receptors, leading to a reduced BBB/BBTB integrity and an elevated drug concentration in cerebral parenchyma (Alyautdin et al., 2014; Jue & McDonald, 2016). In comparison to bradykinin, RMP-7 is not only more potent and B2-selective but also resistant to degradation of bradykinin-metabolizing enzymes. In an RG2 rat-glioma model, the delivery of carboplatin to tumors was improved by 30–80% with co-administration of RMP-7 (Borlongan & Emerich, 2003). Another option for opening the BBB by chemical agents is the utilization of alkylglycerols (AGs). Two short-chain AGs, 1-O-pentylglycerol and 2-O-hexyldiglycerol, showed a reversible increase in BBB permeability without alterations of TJ strand complexity in an *in vitro* BBB model of primary rat brain endothelial cells co-cultured with rat cerebral glial cells (Hülper et al., 2013). Moreover, the intracarotid injection of 200 mM 1-O-pentylglycerol led to a significant increase in brain concentration of the co-injected methotrexate in a concentration-dependent manner in nude mice (Erdlenbruch et al., 2003). Despite the complex nature of chemical-mediated BBB/BBTB disruption, involving limited effectiveness and side effects, this regimen continues to remain an active area of BBB/BBTB breaching due to the BBB/BBTB interactive potential of vasoactive mediators.

Similarly, hyper-osmotic BBB/BBTB disruption refers to a technique that has been widely applied for transiently opening the BBB/BBTB and increasing BBB/BBTB permeability by hyperosmolar agents, most commonly mannitol. Following the administration of mannitol, water was withdrawn from the endothelial cells into the vascular lumen, thus effectively contracting intracellular volume. Dehydration of cerebral endothelium and shriveling of endothelial cells cause a consequent opening of TJs and an increased BBB permeability (Rodriguez et al., 2015). This allows for a therapeutic window of a few hours, which can then be employed for the intra-arterial (*i.a.*) administration of the chemotherapeutic agents, such as methotrexate or carboplatin (Gerber et al., 2014). Generally, a chemotherapeutic agent of interest was *i.a.* delivered following the injection of a standard dose of 1.4 M mannitol (10 mL) over two minutes (Burkhardt et al., 2012). Several studies suggest that the method to disrupt the BBB/BBTB by mannitol increases brain entry of anti-tumor chemotherapeutic agents by 10- to 100-fold compared to administrating the drug alone (Miller, 2002). Remarkable evidence of tumor reduction and prolonged progression-free survival have been reported in a phase I trial in the patients with malignant gliomas after mannitol-induced BBB/BBTB disruption followed by *i.a.* administration of melphalan/carboplatin (Guillaume et al., 2010). Although some studies supported the effectiveness and the safety of this approach (Doolittle et al., 2000), the technical challenges of the procedure, as well as systemic neurological toxicity, (Kemper, Verheij, et al., 2004) limit the feasibility of osmotic disruption technique in pre-clinical and clinical trials.

Being different from the approaches disrupting the BBB/BBTB mediated by chemicals and hyperosmotic agents, thermotherapy-induced BBB/BBTB opening encompasses a variety of physical techniques for generating alterations of the barrier integrity. Most relevant to this strategy are the techniques of focused ultrasound (FUS), radio-frequency microwaves, and laser interstitial thermotherapy (LITT). These techniques induce a mechanical destabilization of TJs in the cerebrovasculature and thus enhance the barrier permeability by producing intracranial hyperthermia. FUS is a noninvasive technique which thermo-mechanically disrupts the BBB/BBTB through transcranial delivery of low-frequency ultrasound waves (Hendricks et al., 2015; Rodriguez et al., 2015). FUS itself is able to thermally ablate the tumor tissue and the heating effect on the area of interest can be enhanced in combination with microbubbles (Kim et al., 2008). More importantly, the simultaneous utilization of microbubbles with low-intensity FUS induces transient opening of TJs by producing shear stress in endothelial cells (VanBavel, 2007) or by activation of signaling pathways modulating the barrier permeability (Jalali et al., 2010), leading to a tumor-located and reversible BBB/BBTB disruption (Sheikov et al., 2004). Typical settings of FUS are repeated ultrasonic exposure bursts at the frequency of 1 Hz at 10 ms used for 20–30 s durations (Rodriguez et al., 2015). Intravenous (*i.v.*) administration of microbubbles has been used for lowing the ultrasound intensity required for BBB/BBTB disruption (Hynynen et al., 2001). Moreover, the size selectivity of BBB/BBTB disruption can be manipulated by the applied FUS, allowing for BBB/BBTB crossing of the agents bearing a molecular weight up to 2000 kD (Chen & Konofagou, 2014). Several pre-clinical studies have approved the feasibility of BBB/BBTB disruption by FUS in the administration of chemotherapeutic agents for GBM therapy. The FUS-induced BBB/BBTB disruption was reported to improve brain accumulation of temozolomide (TMZ) from 6.98 to 19 ng/mg in nude mice bearing human U87 glioma cells (Liu et al., 2014). In another pre-clinical study, cerebrospinal fluid (CSF)/plasma ratio of TMZ was increased from 22.7% to 38.6% by application of FUS in rats with gliomas (Wei et al., 2013). In healthy rabbits, delivery of Irinotecan was elevated from 206% to 331% via FUS-induced disruption technique (Beccaria et al., 2016). Moreover, prolonged survival has been observed in rats with glioma that treated by FUS accompanied by BBB-impermeable liposomal doxorubicin (Aryal et al., 2013). Reportedly, the microbubble-enhanced FUS was shown to promote brain entry of carmustine (BCNU) in rats, as described by Ting et al. (Ting et al., 2012). Despite the risk of uncontrolled thermal injury in brain as well as undesirable side effects of edema and intracerebral hemorrhage, FUS technique displayed several advantages over other approaches for BBB/BBTB disruption because it is noninvasive, repeatable, and economical to conduct. Recently, this technique has been translating from pre-clinical studies to the clinic. In a clinical case, in a patient with recurrent GBM 0.7 cc of the tumor, which corresponds to 10% of the enhancing tumor volume, was ablated following the FUS application of 25 sonications of 10–25 s and 150–950 Watts of acoustic energy used for only 5 h (Coluccia et al., 2014). Therefore, FUS-induced BBB/BBTB disruption coupled with microbubbles seems very feasible in GBM patients in the future clinical trials and shows translational study success to warrant further optimization of this approach in clinical trials.

Radio-frequency microwaves and laser interstitial thermotherapy (LITT) are two additional modalities that induce BBB/BBTB disruption through the administration of heat. The former originates from the typical utility of radiation therapy to ablate the brain tumor by inducing DNA damage. Since a minimal invasive and selective BBB/BBTB disruption by radio-frequency microwaves was seen in several animal and human models (Patel & Mehta, 2007; Lemasson et al., 2010), radiation therapy is expected to be applied in both tumor ablation and BBB/BBTB disruption in the future clinical studies. By contrast, LITT is a novel technique that allows for destroying the tumor tissue via laser ablative hyperthermia (Rodriguez et al., 2015). LITT is able to cause a destruction of cell membranes, consequently leading to coagulative necrosis of the tumor. Meanwhile, cell membrane destruction also induces BBB/BBTB disruption, which may provide a chance for brain entry of anti-tumor agents. To date, LITT has been successfully used for the treatment of a panel of tumors including GBM (Carpentier et al., 2012; Sloan et al., 2013). Importantly, the LITT-induced thermal ablation region where the compromised BBB/BBTB occurs has been observed by followed gadolinium-enhanced magnetic resonance imaging (MRI) (Hawasli et al., 2014). Nonetheless, whether BBB/BBTB crossing of chemotherapeutic agents is enhanced following LITT application remains still unknown.

## 4. Conclusions and prospect

Till date, GBM remains one of the most difficult-to-treat central nervous system tumors, and the most aggressive in nature. The grim prognosis and inevitable recurrence characterize this neoplasm, which is at least in part ascribed to lack of effective brain entry of most anti-tumor chemotherapeutic agents to achieve sufficient accumulation in the invasive zone beyond the bulk GBM. Both BBB and BBTB serve as the major hurdles hampering effective treatment of GBM by denying chemotherapeutic agents reach the tumor infiltration regions. In this review, we have focused exclusively on the delivery of small-molecule anti-tumor agents across the BBB/BBTB as well as advances in development of therapeutic strategies to overcome the BBB/BBTB. Bearing in mind the irreplaceable role of chemotherapy in treatment of GBM, identification of novel small-molecule chemotherapeutic agents with both potent anti-GBM activity and promising brain entry efficacy is always commendable and is in urgent need. Herein we have presented a comprehensive synopsis concerning evaluation of the BBB/BBTB permeability of marketed drugs including temozolomide (the current standard chemotherapy in GBM) and their anti-tumor efficacy *in vivo* as well as a simple drug screening strategy combining the advantages of medicinal chemistry and drug re-purposing. In parallel, opening or disruption of BBB/BBTB also hold the key to enhance the delivery of small anti-tumor agents to the brain. Targeted inhibition of active efflux transporters (AETs) at the BBB/BBTB might be preferred to confer an improved drug delivery. In addition, multiples new chemical and physical therapeutic approaches have been introduced for BBB/BBTB disruption, such as chemical and osmotic disruption technique as well as thermotherapy. Investigation into these new approaches and techniques will provide the fundamental basis for an optimized combinatorial treatment and accordingly abrogate the obstacles that previously hindered brain access of anti-tumor drugs.

As we stand today, GBM remains incurable worldwide and few patients with malignant GBM have yet profited although considerable effort and investment have been devoted to the development of novel chemotherapeutic drugs and new therapeutic approaches for GBM. The battle against lethal GBM demands more powerful weapons and strategies. Continuous efforts deserve to be directed toward medicinal chemistry to discover more potent small-molecule chemotherapeutic agents and toward advancing the current approaches for BBB/BBTB disruption to clinical applications in GBM patients. Convincingly, a growing number of small anti-GBM drugs bearing superior BBB/BBTB-penetrating characteristics as well as innovative strategies and techniques breaching the BBB/BBTB are going to emerge in the foreseeable future and to give us breakthrough news. Hopefully, stepwise advancements of therapeutic benefit can be approved in a few years ahead.

## Supplementary Material

Supplemental_Material-2.docx

Supplemental_Material-1.docx
