# Supplementary material for: A comprehensive review in improving delivery of small-molecule chemotherapeutic agents overcoming the blood-brain/brain tumor barriers for glioblastoma treatment
Source: Drug Deliv. 2019 May 16;26(1):551–65. doi: 10.1080/10717544.2019.1616235 (PMC6534214; doi:10.1080/10717544.2019.1616235)
Supplement: Supplemental_Material-2.docx [file IDRD_A_1616235_SM1484.docx]

**Table S2** BBB/BBTB- or BBB-penetration related parameters of compound **1** to **16** potentially for GBM treatment, the prediction of the rule of 5 and their BBB/BBTB or BBB penetration status *in vivo*

| Compound | MW/Da | Log P | Hydrogen bond donor count | Hydrogen bond acceptor count | PSA/nm^2^ | Rule of 5 | BBB/BBTB or BBB penetration (Ref.)^*^ |
| --- | --- | --- | --- | --- | --- | --- | --- |
| **1** | 343 | 1.5 | 2 (NH) | 5 | 0.70 | Yes | + (Yap et al., 2011) |
| **2** | 465 | NA | 2 (NH) | 7 | 0.78 | Yes | + (Beyer et al., 2008) |
| **3** | 353 | 4.5 | 2 (NH+OH) | 3 | 0.45 | Yes | + (Hammarström et al., 2016) |
| **4** | 492 | 7.1 | 1 (NH) | 5 | 0.54 | No | + (Kim et al., 2015) |
| **5** | 561 | 2.6 | 3  (2NH+OH) | 9 | 1.07 | No | + (Norman et al., 2012) |
| **6** | 518 | 2.0 | 2 (NH) | 12 | 1.50 | No | + (Chen et al., 2014) |
| **7** (Trifluoperazine) | 408 | 4.9 | 0 | 3 | 0.10 | Yes | + (Pinheiro et al., 2017) |
| **8** (Quinacrine) | 400 | 6.7 | 1(NH) | 4 | 0.37 | No | + (Golden et al., 2015) |
| **9** (dacomitinib) | 470 | 5.7 | 2 (NH) | 7 | 0.78 | No | + (Zahonero et al., 2015) |
| **10** (Pyrvinium) | 383 | 2.8 | 0 | 3 | 0.09 | Yes | + (Venugopal et al., 2015) |
| **11a** | 237 | 1.7 | 2 (NH) | 5 | 0.63 | Yes | + (Łazewska et al., 2009) |
| **11b** | 237 | 1.5 | 2 (NH) | 5 | 0.63 | Yes | + (Łazewska et al., 2009) |
| **12** | 377 | 4.2 | 0 | 5 | 0.48 | Yes | + (Ishikawa et al., 2010) |
| **13** | 418 | 5.9 | 1(NH) | 5 | 0.63 | No | + (Chiriano et al., 2012) |
| **14** | 381 | 4.8 | 0 | 5 | 0.44 | Yes | + (Wager et al., 2010; Asproni et al., 2011) |
| **15** | 420 | 4.3 | 2 (NH) | 5 | 0.52 | Yes | + (Caliş et al., 2011) |
| **16** | 350 | 3.2 | 1 (NH) | 6 | 0.46 | Yes | + (Butini et al., 2009) |

* BBB/BBTB penetration *in vivo* is shown here for compound **1** to **10** and BBB penetration *in vivo* for compound **11** to **16**.
