# Supplementary material for: A comprehensive review in improving delivery of small-molecule chemotherapeutic agents overcoming the blood-brain/brain tumor barriers for glioblastoma treatment
Source: Drug Deliv. 2019 May 16;26(1):551–65. doi: 10.1080/10717544.2019.1616235 (PMC6534214; doi:10.1080/10717544.2019.1616235)
Supplement: Supplemental_Material-1.docx [file IDRD_A_1616235_SM1483.docx]

**Table S1** BBB/BBTB-penetration related parameters of approved small chemotherapeutic agents for GBM, the prediction of the rule of 5 and their BBB/BBTB penetration status *in vivo*

| Chemotherap-eutic agents | MW/Da | Log P^*^ | Hydrogen bond donor count | Hydrogen bond acceptor count | PSA/nm^2^ | Rotatable bond count | Protein binding | Rule of 5 | BBB/BBTB penetra-tion | B/P  ratio (Ref.)^**^ |
| --- | --- | --- | --- | --- | --- | --- | --- | --- | --- | --- |
| Temozolomide (TMZ) | 194 | -2.8 | 1 | 5 | 1.06 | 1 | 15% | Yes | + | 18%(H)(Reyderman et al., 2004);22-41%(R) (Ostermann et al., 2004) |
| Lomustine (CCNU) | 234 | 2.8 | 1 | 2 | 0.62 | 4 | 50% | Yes | + | 20%(R) (Jr et al., 1980) |
| Carmustine (BCNU) | 214 | 1.5 | 1 | 2 | 0.62 | 5 | 80% | Yes | + | 30%(R) (Dréan et al., 2016) |
| Procarbazine | 221 | 0.1 | 3 | 3 | 0.53 | 5 | NA | Yes | + | Medium (R) (Dréan et al., 2016) |
| Cisplatin | 298 | -2.2 | 2 | 2 | 0.52 | 0 | 90%^***^ | Yes | + | 10%(R) (Jacobs et al., 2010) |
| Carboplatin | 371 | NA | 0 | 0 | 1.08 | 0 | 90%^***^ | Yes | + | NA(H/R) |
| ACT001 | 409 | 1.5 | 1 | 4 | 0.50 | 1 | NA | Yes | + | 50-55.6%(R) (An et al., 2015) |
| Topotecan | 421 | 0.8 | 2 | 6 | 1.03 | 3 | 35% | Yes | - | 5.5%(R) (Dréan et al., 2016) |
| Methotrexate | 454 | -1.9 | 5 | 12 | 0.21 | 9 | 50% | No | - | 3-21%(R) (Westerhout et al., 2014) |
| Doxorubicin | 544 | 1.3 | 6 | 12 | 0.21 | 5 | 74-76% | No | - | 0-0.5%(R) (Van et al., 1999) |
| Etoposide | 589 | 0.6 | 3 | 12 | 0.16 | 5 | 97% | No | - | 3-8%(R) (Dréan et al., 2016) |
| Irinotecan | 587 | 3.2 | 1 | 6 | 0.11 | 5 | 30-68% | No | + | 9-13%(R) (Goldwirt et al., 2014) |
| Vincristine | 825 | 2.8 | 3 | 9 | 0.17 | 10 | ~75% | No | - | NA(H/R) |

* Log P, the experimental Log P; ** B/P ratio, the normal brain/plasma ratio of drugs in human (H) or in rats (R); *** 90%, 90% free platinum. All the parameters presented here collected from the public data sources of drugbank (<https://www.drugbank.ca/drugs>).
